# Current Advances in *Burkholderia* Vaccines Development

**DOI:** 10.3390/cells9122671

**Published:** 2020-12-11

**Authors:** Guanbo Wang, Paulina Zarodkiewicz, Miguel A. Valvano

**Affiliations:** Wellcome-Wolfson Institute for Experimental Medicine, Queen’s University Belfast, Belfast BT9 7BL, UK; G.Wang@qub.ac.uk (G.W.); P.Zarodkiewicz@qub.ac.uk (P.Z.)

**Keywords:** *Burkholderia mallei*, *Burkholderia pseudomallei*, *Burkholderia cepacia* complex, DNA vaccines, whole-cell vaccines, liver attenuated vaccines

## Abstract

The genus *Burkholderia* includes a wide range of Gram-negative bacterial species some of which are pathogenic to humans and other vertebrates. The most pathogenic species are *Burkholderia mallei*, *Burkholderia pseudomallei*, and the members of the *Burkholderia cepacia* complex (Bcc). *B. mallei* and *B. pseudomallei*, the cause of glanders and melioidosis, respectively, are considered potential bioweapons. The Bcc comprises a subset of *Burkholderia* species associated with respiratory infections in people with chronic granulomatous disease and cystic fibrosis. Antimicrobial treatment of *Burkholderia* infections is difficult due to the intrinsic multidrug antibiotic resistance of these bacteria; prophylactic vaccines provide an attractive alternative to counteract these infections. Although commercial vaccines against *Burkholderia* infections are still unavailable, substantial progress has been made over recent years in the development of vaccines against *B. pseudomallei* and *B. mallei*. This review critically discusses the current advances in vaccine development against *B. mallei*, *B. pseudomallei,* and the Bcc.

## 1. Introduction

The genus *Burkholderia* comprises a wide range of environmental Gram-negative bacteria that interact with plants, insects, animals, and humans. Most of the species in the genus are plant-associated bacteria [1]. However, some species are also dangerous opportunistic pathogens to animals and humans [1]. The most clinically relevant species include *Burkholderia mallei*, *Burkholderia pseudomallei,* and the members of the *Burkholderia cepacia* complex (Bcc). All *Burkholderia* are intrinsically resistant to a wide range of antibiotics, which complicates the treatment of their infections; prophylactic vaccines are an attractive alternative to protect against *Burkholderia* infections.

*B. mallei* and *B. pseudomallei* are phylogenetically similar; they cause glanders and melioidosis, respectively, and have been classified as Tier 1 select agents by the US Centers for Disease Control and Prevention (https://emergency.cdc.gov/agent/agentlist-category.asp). *B. mallei* is a facultative intracellular pathogen generally infecting horses, mules, and donkeys, and sporadically humans [2]. Glanders may present either as an acute or chronic infection, depending on the infection route, with high fever, weight loss, malaise, abscess formation, pneumonia, and septicemia [3]. Although glanders has been successfully eliminated from North America, Australia, and Europe, recent equid outbreaks in the Middle East and Asia pose a threat for reintroducing the disease into disease-free areas [4,5,6,7,8]. *B. pseudomallei*, a saprophyte commonly present in wet soils and rice paddies, is endemic in tropical and subtropical countries [9]; it is a leading cause of sepsis in Northern Australia [10] and bacterial pneumonia in Thailand [11]. Epidemiological modeling suggests that the incidence of melioidosis is underestimated in countries which are known to be endemic and is predicted to extend to many countries where the disease was not previously reported [9]. *B. mallei* and *B. pseudomallei* are resistant to broad-spectrum antibiotics including aminoglycosides, polymyxins, and β-lactams [12]. Both glanders and melioidosis have high mortality rates (up to 50%) even despite aggressive antimicrobial therapy [11].

The Bcc encompasses a subgroup of *Burkholderia* species that cause respiratory infections in people with underlying diseases, such as cystic fibrosis and chronic granulomatous disease [13]. The Bcc consists at least 24 genetically closely related species [14,15,16,17,18]; the most common species found in people with cystic fibrosis are *B. cenocepacia*, *B. multivorans*, *B. vietnamiensis*, *B. dolosa,* and *B. cepacia* [19]. *B. cenocepacia* and *B. multivorans* are highly prevalent, as they account for approximately 85–97% of all Bcc infections in people with cystic fibrosis [20]. Treatment of Bcc infections is challenging, as therapies with antibiotics are often less effective among chronically infected patients due to the intrinsically high level of antibiotic resistance of these pathogens [19]. Developing effective vaccines to confer broad protection against Bcc infections is a desired alternative, but very few studies on Bcc vaccines have been reported to date [21,22,23,24].

Substantial advances have been made over the past decade in understanding the pathogenesis, host–pathogen interactions, virulence factors, and host immune responses against *Burkholderia* species. However, no ideal vaccine candidate appeared for humans, reflecting the challenges in designing safe and effective vaccines that can elicit long lasting immunity to protect against both acute and chronic infections by these bacteria. Here, we provide a critical overview of recent advances in *Burkholderia* vaccine development and how new technologies could drive the design of effective vaccines against *Burkholderia* infections.

## 2. *Burkholderia* Vaccine Development Strategies

### 2.1. Inactivated Whole-Cell Vaccines

Inactivated whole-cell vaccines contain bacteria that have lost the capacity to cause disease. Heat, chemical, and UV radiation are the most common methods to inactivate bacteria; the primary advantage of inactivated whole-cell vaccines is their ability to induce robust humoral immune responses. Nonreplicating, inactivated vaccines have been licensed against 11 different human pathogens including viruses and bacteria [25], such as for example *Bordetella pertussis* and *Neisseria meningitidis* serogroup B infections [25].

Although inactivated whole-cell vaccines against Bcc species have not been reported, several groups have developed inactivated *B. pseudomallei* vaccines (Table 1), illustrating how the mode of inactivation and administration influence vaccine efficacy [26,27,28,29]. Vaccination with heat-killed *B. pseudomallei* induced high levels of protection against subsequent homologous *B. pseudomallei* challenge in mice [26]. Additionally, immunization with heat-killed *B. mallei* and *B. thailandensis* conferred some cross protection against *B. pseudomallei* infection challenge [26]. Cross-reactive antibodies elicited by conserved antigens may result in cross protection among *B.* mallei, *B. pseudomallei,* and *B. thailandensis* since the genomes of these species are similar [30]. Mucosal immunization with heat-killed *B. pseudomallei* mixed with cationic liposomes complexed with noncoding plasmid DNA as the mucosal vaccine adjuvant, effectively elicited mucosal IgA and systemic IgG responses, and stimulated antigen-specific CD8^+^ in the airways. This vaccine conferred significant protection against lethal pulmonary *B. pseudomallei* challenge in mice, as demonstrated by 100% survival of vaccinated mice for > 40 days [31], suggesting that immunization through aerosol route may be more effective than the intraperitoneal route for inducing mucosal immunity.

The protectivity of paraformaldehyde-killed (PP) and heat-killed (HK) *B. pseudomallei* together with CpG ODN adjuvant has been compared; PP bacteria provided higher protection than HK against intraperitoneal challenge with *B. pseudomallei* in mice, as PP-immunized mice survived significantly longer than HK-immunized mice [29]. It remains unclear why the PP preparation elicited better protection than HK bacteria. The PP vaccine showed fewer protein bands in silver-stained SDS-PAGE gel when compared to the HK counterpart, and a protein with molecular weight of 30 kDa in PP bacteria reacted with convalescent but not acute mouse sera. Therefore, the 30-kDa protein present in the PP vaccine may be required to induce a protective immune response [29]. A similar observation was made concerning the antigenicity and immunogenicity of *Vibrio cholerae*, which are significantly affected by the inactivation method used for vaccine production [32]. Therefore, inactivation conditions are important factors that could affect the efficacy of killed bacteria as vaccine antigens.

For *B. mallei*, heat-killed and UV-inactivated wild type and a capsule-negative mutant were evaluated in mice as protective vaccines. Immunized mice generated mixed Th1 and Th2 cytokine responses and a Th2-biased immunoglobulin response, respectively; however, these preparations did not protect mice against live challenge with *B. mallei* [27]. Similar results were reported in a subsequent study, where vaccination with heat-killed *B. mallei* only conferred partial protection of mice from lethal *B. mallei* challenge [33]. B cells, CD4^+^, and CD8^+^ T cells, as well as IFN-γ and TNF-α cytokines, appear to play important roles in the protection mediated by the heat-killed *B. mallei* vaccine [33].

Collectively, although some protection against *B. mallei* and *B. pseudomallei* challenge was observed in mice immunized with inactivated whole-cell vaccines, immunized mice were incompletely protected. Several factors, such as inactivation conditions, choice of adjuvants, and immunization route must be considered when evaluating the protective efficacy of an inactivated *Burkholderia* vaccine.

### 2.2. Live Attenuated Vaccines

The best example of a successful live attenuated bacterial vaccine is a licensed vaccine against *Salmonella enterica* serovar Typhi [34], which is based on the Ty21a strain constructed in the early 1970s using chemical mutagenesis [35]. Attenuated bacteria are commonly generated by mutating genes encoding proteins essential for cellular metabolism/transport pathways, secretion systems, and pathogenesis.

By transposon mutagenesis and genome sequencing, various essential genes have been identified in *Burkholderia* species [36] and the live attenuated vaccines derived from this information are summarized in Table 2. An auxotrophic mutant (Δ*ilvI*) defective in the branched chain amino acid biosynthetic pathway provided significant protection in mice against semi lethal doses of wild-type *B. pseudomallei* [37]. CD4^+^ and CD8^+^ T cell depletion studies showed that the protection was mediated by CD4^+^ T cells [38]. Other mutants, defective in purine biosynthesis (Δ*purN*, Δ*purM*) and additional biosynthetic pathways, were also tested as candidate attenuated vaccines [36]. Challenge experiments demonstrated that mice vaccinated with Δ*purN* strains either by intranasal or intraperitoneal routes were protected against acute infection while no protection was observed against intravenous challenge. However, mice infected by intranasal or intraperitoneal routes eventually showed clinical signs of disease after 20 days or more post-challenge and succumbed from chronic infection [39]. It is possible that the purine biosynthetic pathway may not be completely interrupted in the Δ*pur*N mutant making it not fully attenuated [36]. In contrast, the Δ*purM* mutant derived from strain 1026b, which is defective in adenine and thiamine biosynthesis, has been proven to be fully attenuated and safe for use under BSL-2 laboratory conditions [40,41]. Despite these differences between Δ*purN* and Δ*purM* mutants, the results using them as vaccine antigens are inconclusive [36,39].

A set of two auxotrophic mutants in the biosynthesis of aromatic compounds, Δ*aroB* and Δ*aroC*, have also been evaluated as attenuated vaccines [42,43]. Although significant delay in time to death was observed in mice immunized with the Δ*aroB* strain, all mice eventually died. Further, immunization with the Δ*aroC* mutant protected C57Bl/6 mice but not BALB/c mice from infection challenge [43]. It was speculated that the Δ*aroC* mutant is too attenuated to develop a protective immune response in BALB/c mice since it is rapidly cleared.** A deletion mutant, Δ*asd*, auxotrophic for diaminopimelate in rich medium and auxotrophic for diaminopimelate, lysine, methionine, and threonine in minimal medium, has been evaluated for protective efficacy as a live attenuated vaccine [44]. This vaccine significantly increased mice survival after challenge but did not protect mice from chronic infection [44]. Another study investigated the protective effect of a double mutant Δ*relA*Δ*spoT*, which lacks (p)ppGpp-synthesizing enzymes [45]. Vaccination of mice with the Δ*relA*Δ*spoT* mutant provided significantly increased survival (100% survivors) up to 30 days post-challenge; however, sterilizing immunity was not achieved.

Due to their importance in bacterial pathogenicity, protein secretion systems have been exploited to develop attenuated vaccines against *B. pseudomallei* infection. The *B. pseudomallei* Δ*bipD* mutant, lacking a putative protein associated with the translocation apparatus of the type 3 secretion system, was significantly attenuated in BALB/c mice [46]. However, this mutant cannot prevent fatal melioidosis. Prior infection with Δ*bipD* partially protected mice (60% survival) from infection with the wild-type *B. pseudomallei* challenge. However, immunization with the BipD protein alone failed to confer protection, suggesting BipD is not a protective antigen for *B. pseudomallei* [46].

Similar strategies have been adopted to generate attenuated *B. mallei* strains. The attenuated *B. mallei* strains Δ*ilvI* [47], Δ*tssN* [48], Δ*tonB* [49], and Δ*tonB*Δ*hcp1* [50] have been tested in mice. Vaccination with Δ*ilvI* provided partial protection in BALB/c mice against high-dose aerosol challenge with *B. mallei* ATCC23344. The surviving mice, however, developed chronic infection, suggesting the vaccine was not efficient to elicit enough protection to eliminate all bacteria in the inoculum or alternatively, had no effect on bacteria internalized in host cells [47]. Vaccination with Δ*tssN* provided some protection against aerosol challenge with high-dose ATCC23344 in BALB/c mice, but surviving mice experienced significant weight loss indicative of chronic infection [48]. Vaccination with Δ*tonB* conferred 100% protection to BALB/c mice against aerosol challenge with the wild type strain CSM001. However, this mutant may not have been fully attenuated, as the immunized mice developed splenomegaly and multiple splenic abscesses [49]. The Δ*tonB*Δ*hcp1* double mutant appears to be the safest and most effective *B. mallei* and *B. pseudomallei* attenuated vaccine tested so far (Table 2); complete protection was observed in immunized mice with no pathological lesions and minimal residual bacterial numbers in organs after infection challenge [50,51]. Vaccination of mice generated robust humoral and cellular immune responses; however, CD4^+^ and CD8^+^ T cells did not appear to be critical for protection [50,51]. Zimmerman et al. [52] investigated the protective effect of another attenuated mutant, Δ*batA*, against *B. mallei* infection in mice. BatA is an autotransporter protein that has been reported to be selectively expressed in vivo or in vitro under conditions that mimic the host environment, indicating the protein is important for bacterial intracellular survival. Their results showed that vaccination with Δ*batA* mutant provided significant protection against aerosol challenge with lethal doses of wild type strains of *B. mallei* and *B. pseudomallei* in mice [52].

There is only one live-attenuated vaccine for the Bcc, which has been tested in a mouse infection model [21]. The attenuated strain was generated by mutating the *tonB* gene in the *B. cenocepacia* K56-2 strain and could confer significant protection in mice against acute respiratory *B. cenocepacia* lethal infection with a survival rate of 87.5% at day 6 post infection.

Further investigation of protectivity against chronic infection of melioidosis and glanders is needed, especially because it is unclear if the results using mice models are extrapolatable to humans. Both *B. pseudomallei* and Bcc species can establish chronic infections in humans. A chronic *B. pseudomallei* infection can be manifested as a systemic illness that lasts longer than 2 months in patients, while in the case of Bcc infections, chronic pneumonia is also frequently observed in people with cystic fibrosis and chronic granulomatous disease [53]. Therefore, models that can mimic the chronic infection in humans are essential to evaluate the long-term protective efficacy of these vaccines. The murine models of chronic infection for anti-*Burkholderia* vaccine development have been recently reviewed elsewhere [53,54,55].

### 2.3. Subunit Vaccines

Despite the promise held by live attenuated vaccines based on *B. pseudomallei* mutants, they also pose safety concerns. In contrast, subunit vaccines containing one or more antigens or epitopes are deemed to be safer and potentially nonreactogenic. Various antigens have been identified for subunit vaccines against *Burkholderia* species [23,56,57,58,59]. *Burkholderia* subunit vaccines have been tested in mice and Rhesus macaques (Table 3) but so far, they have not advanced to clinical trials. Many of the antigens chosen for these vaccines are conserved among multiple strains of *B. pseudomallei* and *B. mallei* and include molecules that have major roles in virulence.

LolC, a component of the lipoprotein export system from the inner to the outer membrane in Gram-negative bacteria, is a proposed candidate for a subunit vaccine targeting *B. mallei* and *B. pseudomallei* strains. This strongly seroreactive protein can induce lasting immune memory, an essential attribute of a successful vaccine. The protein is also conserved across the Bcc; however, it has not been tested in a Bcc infection model. LolC stimulated *B. pseudomallei*-specific humoral and cellular responses, granting significant protection to subsequent infection; the protein was also more protective when administered subcutaneously with immune stimulating complexes, such as CpG oligodeoxynucleotide 10103, an inoculation route that is more compatible with clinical applications [60]. Similarly, immunization with *B. pseudomallei* LolC conferred protection against inhaled infection by *B. mallei* [61]. Cross-protection was also reported upon immunization with *B. pseudomallei* BopA, a type III secreted protein, resulting in 100% and 60% survival against *B. mallei* and *B. pseudomallei*, respectively [61].

Outer membrane proteins are surface antigens also considered for vaccine development [57]. Immunization with *B. cenocepacia* OmpW, a protein involved in attachment to host epithelial cells, lowered *B. cenocepacia* and *B. multivorans* burden in lungs, and was a protective antigen for mice challenged with either species [23]. OmpW is conserved across 13 sequenced *B. pseudomallei* strains, suggesting OmpW could protect against multiple strains [65]. Indeed, *B. pseudomallei* OmpW, together with SAS adjuvant, significantly enhanced survival in two mouse models (75% C57Bl/6 mice survival at day 80) demonstrating an efficiency greater than that of the live attenuated 2D2 positive control [65]. The OmpA-like protein protects from pulmonary colonization in mice; when co-administered with a mucosal nanoemulsion adjuvant, shows a cross-neutralizing immunity against *B. cenocepacia* and *B. multivorans* and a balanced Th1/Th2 immune response [69]. Two OmpA domains, Omp3 and Omp7, induced protection in 50% of mice [63]. Further*, B. cenocepacia* OmpA specific antibodies are present in sera of people with cystic fibrosis, indicating its potential to naturally stimulate humoral responses [56]. Another outer membrane protein, Omp85, can induce protection in mice from bacterial challenge, but it mainly stimulated a Th2 immune response [62].

Other virulence factors, such as proteins associated with the type 6 secretion system and trimeric autotransporter adhesins have been investigated as subunit vaccine candidates. The *B. pseudomallei* hemolysin-coregulated protein Hcp2 provided 80% protection to challenged mice, while Hcp1, Hcp3, and Hcp6 provided 50% protection [68]. Anti-Hcp1 specific antibodies (IgM and IgG) are present in sera of melioidosis patients, suggesting that humans develop a humoral response to this antigen [70]. Trimeric autotransporter adhesins, like PSL2063 and BimA, have also been described for their immunogenic properties in relation to *B. pseudomallei* and *B. mallei* [61,71,72]. Antigenic protein profiling of *B. pseudomallei* using a goat model of melioidosis identified multiple antigens. From these, the GroEL heat shock protein 60, EF-Tu, ATP synthase β chain, and the DnaK chaperone provided the strongest immune responses, suggesting they could be good candidates for further investigation [73].

Outer membrane vesicles have also been tested as vaccines against *B. pseudomallei* and *B. mallei* in both mice and Rhesus macaques. Immunization of mice with *B. pseudomallei* outer membrane vesicles elicited humoral and cellular immune responses against the bacterium [66,67]. In Rhesus macaques, outer membrane vesicles induced humoral responses to protective protein and polysaccharide antigens, without any toxicity and reactogenicity; they also induced significant protection against *B. mallei* infection, reflected by production of *B. mallei*-specific serum IgG in both mice and Rhesus macaques, and *B. mallei*-specific Th1/Th14 CD4^+^ and CD8^+^ T cell responses in mice [66,67,74].

Reverse vaccinology strategies based on bioinformatic pipelines have opened new possibilities to identify novel vaccine candidates. They can be used to predict new antigens, such as the *B. pseudomallei* type I fimbria subunit BPSL1626, which can recognize and bind antibodies in sera from infected patients and induce T-lymphocyte responses in vivo [75]. Three epitopes have been predicted, which could be used as peptides to elicit enhanced immunity, as previously shown for the BPSL2765 antigen [76]. Reverse vaccinology, together with subtractive genomics, has also been used for the identification of more than 60 *B. pseudomallei* (Bp1651) proteins as potential vaccine targets [77].

Due to the intracellular survival of *Burkholderia* and the heterogeneity of the genus a multicomponent subunit vaccine is likely required to induce a balanced immune response resulting in long-lasting immunity. So far, no ideal vaccine candidate has been identified. The current candidates only offer sterilizing immunity at relatively low challenge doses, which may be insufficient for a fully protective vaccine. However, this low protection threshold could still be effective to reduce the incidence of disease and several vaccine formulations may become useful with additional optimization.

### 2.4. Glycoconjugate Vaccines

Polysaccharides are attractive candidate antigens for vaccine development, as they contain unusual carbohydrates that can be highly antigenic [78,79,80]. A typical glycoconjugate vaccine encompasses a polysaccharide antigen covalently linked to a protein carrier. Carbohydrates are classical T-cell independent antigens typically unable to induce a long-lasting T-cell memory. Linking polysaccharides to a protein allows these antigens to be processed through a T-cell-dependent pathway, which facilitates recall responses associated with long-term immune memory. Bacterial capsular polysaccharides (CPS), lipopolysaccharides, exopolysaccharides [81], or O-antigens are common carbohydrate candidates for producing glycoconjugate vaccines (Table 4).

CPS is a recognized virulence factor for both *B. pseudomallei* and *B. mallei* [82,83]. Immunization with *B. pseudomallei* CPS covalently linked to diphtheria toxin mutant (CRM) induces high IgG titers and opsonizing antibodies in C57BL/6 mice [84]. When combined with Hcp1, a component of the T6SS, CPS can induce interferon gamma secretion from T cells, resulting in 100% survival of animals challenged with *B. pseudomallei* [84]. Further, up to 70% of challenged mice showed no culturable *B. pseudomallei* in lungs, spleen, and liver, indicating the vaccine provides sterilizing immunity [84]. Other carrier proteins such as bovine serum albumin (BSA) and LolC, conjugated with *B. pseudomallei* CPS, also provided robust protection [85]. Concerning LolC, which unlike albumin is also a vaccine antigen, the level of protection was higher with the conjugate vaccine than with each component individually [85]. Synthetic production of unbranched CPS consisting of 1→3 linked 2-*O*-acetyl-6-deoxy-β-d-*manno*-heptopyranose, coupled to the tetanus toxin Hc domain (TetHc), elicited IgM and IgG antibodies recognizing the native capsule and protected mice from challenge with *B. pseudomallei* [86].

Lipopolysaccharide (LPS) linked to carrier proteins is another strategy used for production of glycoconjugate vaccines. TetHc, Hcp1, and FliC are commonly used as carriers for *Burkholderia* glycoconjugates, and they can be packed into nanoparticles to improve efficacy by promoting antigen presentation [84,87,88]. Gold nanoparticles are of special interest because of their optical properties for imaging and site-directed release of antigens [88,89,90,91,92]. Protein carriers can be coupled with gold nanoparticles followed by conjugation to purified LPS. This technique has been implemented to develop a vaccine candidate against *B. mallei*, using LPS from *B. thailandensis*, which has been tested in mice and Rhesus macaques [87,89].

The potential of exploiting the *N*- and *O*-glycosylation machineries for the production of glycoconjugates has also been examined [94]. The *N*-glycosylation system of *Campylobacter jejuni* can be functionally reconstructed in *Escherichia coli* to express the periplasmic AcrA protein glycosylated with *B. pseudomallei* O-polysaccharide [93]. The *O*-glycosylation cluster, common to all *Burkholderia* species, can also be potentially exploited to produce recombinant glycoprotein-based vaccines [95,96]. Glycoconjugate vaccine design has been influenced by advances in reverse vaccinology [97]. In silico methods can be used to identify protein candidates by analyzing subcellular location, transmembrane domains, and ability to interact with MHC I and II [97]. This process shows great potential, with reports of in silico identified FlgL which, when linked to gold nanoparticles and *B. pseudomallei* LPS, resulted in 100% mice survival and lower lung colonization after a lethal *B. pseudomallei* challenge [97].

Glycan antigens have proven to be safe and effective, as shown by the licensed glycoconjugate vaccines for *N. meningitidis, S. pneumoniae,* and *H. influenzae*. Candidate *Burkholderia* glycoconjugate antigens (summarized in Table 4) are promising, and their production could be accelerated by novel glycoengineering and glycochemistry tools improving their manufacture.

### 2.5. DNA Vaccines

In contrast to live attenuated vaccines, DNA vaccines have several advantages concerning safety, ease of manufacturing, and stability; they can also induce both long-lasting cellular and humoral immune responses [98]. Although no DNA vaccines are still approved for use in humans, some DNA vaccines have been approved for veterinary use, which include a vaccine against Nile virus in horses and the canine melanoma [99]. Several DNA vaccines have been tested to prevent bacterial infections and the effect of their toxins. Gu et al. reported a DNA vaccine encoding the immunogenic and biologically active portion of anthrax protective antigen (PA), which protected 87% mice from lethal challenge with anthrax toxin [100]. A DNA vaccine for tuberculosis provided some protection to infected animals, as indicated by recovery of Th1/Th2 balance and significant reduction of the pathology [101].

DNA vaccines have also been reported for melioidosis (Table 5) [64,102,103]. Immunization with a CpG-modified DNA plasmid expressing recombinant FliC (flagellin protein) conferred significant protection against intravenous challenge of *B. pseudomallei*, with 93% of immunized mice surviving at 14 days post infection [64]. A subsequent study showed that a modified plasmid, pVAX-hTPA-FliC, elicited strong anti-FliC antibodies and significantly reduced the bacterial load in the lung and pulmonary concentrations of IL-6, CXCL1, and TNF-α at 72-h postinfection [103]. Intranasal immunization using this DNA vaccine protected 53% of mice from intranasal *B. pseudomallei* challenge at 14 days postimmunization [103]. Notably, a comparison of FliC specific antibodies from serum samples of melioidosis-positive patients showed that IgG production is higher in diabetic than in nondiabetic patients [104]. This could be important since diabetes is a risk factor for *B. pseudomallei* infection. However, the variable magnitude of the immune response should be considered when using FliC as a vaccine candidate.

Collectively, although only modest protection can be achieved, DNA vaccines merit further investigation on their protective efficacy against *Burkholderia* infections by testing other potential candidates and delivery systems which can enhance immunogenicity. Further, heterologous prime-boost strategies involving priming with naked DNA followed by boosting with a viral vector expressing the same antigen may enhance vaccine immunogenicity. This strategy has been applied to develop DNA vaccines against different pathogens including tuberculosis; prime-boost BCG vaccination with a lentiviral vector expressing the antigens Ag85B and Rv3425 significantly enhanced immune responses, including T helper type 1 and CD8^+^ cytotoxic T lymphocyte responses, compared with DNA- and protein-based vaccines [105]. Therefore, this approach could be adopted for anti-*Burkholderia* vaccines development and thus warrants additional experimentation.

### 2.6. Viral Vector-Based Vaccines

The concept of viral vector-based vaccines is to deliver one or more antigens encoded in the context of an unrelated and modified virus (attenuated or nonreplicating). Viral vectors have some limitations such as the risk of cancer induced by viral genome being integrated into host genome and the potential of recombinant virus vector being rapidly eliminated by pre-existing immunity. However, viral vectors based on recombinant viruses have been used to develop new vaccines against a wide range of diseases including cancer, human immunodeficiency virus, and malaria [106,107,108]. Adenovirus is one of the most extensive studied viral vectors for vaccine development; it can induce a robust immune response including CD8^+^ cytotoxic T lymphocytes against foreign expressed antigens without the viral genes being integrated into the host genome [109,110]. Numerous viruses, such as retrovirus and lentivirus, have also been exploited as vectors for vaccine development as well as a viral vector-based vaccine against *Brucella melitensis* [111]. The anti-*B. melitensis* vaccine, based on influenza virus, can induce robust B- and T-cell responses and significant protection against *Brucella* infection in pregnant sheep and goats.

The potential of Parainfluenza virus 5 as a vector for vaccine development against glanders and melioidosis was also investigated [112]. Vaccination with a single dose of recombinant Parainfluenza virus 5 expressing BatA (an autotransporter protein) afforded significant protection against aerosol challenge with a lethal dose of *B. mallei* and *B. pseudomallei* (with 74% and 60% immunized mice surviving from chronic infection, respectively; Table 6) [112]. The level of protection against aerosol infection by both *B. mallei* and *B. pseudomallei* using only one dose of a single-antigen vaccine has not been reported.

Despite their limitations, viral vectors exhibit good potential for applications in gene therapy and vaccine development. Concerning viral vector vaccines against *Burkholderia* infections, efforts should be made to search for better vaccine candidate coupled with optimal viral-vector platforms and vaccination strategies.

## 3. Conclusions

While significant progress has been achieved in experimental vaccines to combat *B. pseudomallei* and *B. mallei* infections, no ideal candidate has emerged for use in humans. Concerning Bcc vaccines, a consistent difficulty limiting progress is the lack of proper murine models mimicking chronic human infection since Bcc strains are generally rapidly cleared by the most commonly used mouse strains. Although several gene knock out mice such as chronic granulomatous disease- and cystic fibrosis-deficient mouse models have been developed [55], these models cannot fully replicate the pathophysiology of Bcc infections in humans, posing a strong limitation to test vaccine efficacy. As proposed elsewhere [55], other small animal models, such as *Cftr*−/− ferrets or *Cftr*−/− pigs, could be employed to test vaccine efficacy. Alternatively, a type 6 effector deficient mutant derived from *B. cenocepacia*, Δ*tecA*, was reported to become virulent and lethal to normal mice [113]. The TecA protein can activate the pyrin inflammasome through deamidating a conserved asparagine in Rho GTPase. The detection of TecA by the pyrin inflammasome protects mice from lethal *B. cenocepacia* infection. Therefore, the Δ*tecA* mutant could be used as a strain background to establish a more robust mouse infection model to evaluate vaccine efficacy for Bcc bacteria.

Another challenge for developing anti-*Burkholderia* vaccines is that the vaccine should be able to generate protective immunity in immunocompromised patients. *Burkholderia* species are facultative intracellular pathogens and can establish persistent, chronic infections that can last years. This is demonstrated by the difficulty to demonstrate sterilizing immunity in practically all anti-*Burkholderia* vaccines developed. Murine models of melioidosis have shown CD4^+^ but not CD8^+^ cells to be important in immunity; however, immunological studies in patients have shown that CD4^+^ and CD8^+^ IFN-γ-producing cells are associated with survival [38,114]. Although CD4^+^ responses appear to be important for clearing *Burkholderia* infections, studies at the cellular level show that antibodies alone are not sufficient to clear infection from macrophages, while IFN-γ helps to eliminate the intracellular infection [115]. Therefore, both antibodies and cellular immune responses appear critical from a successful anti-*Burkholderia* vaccine. Future research efforts should identify correlates of immunity that include the elimination of the intracellular infection, which may become a reservoir for surviving bacteria, especially in immunocompromised individuals.

Concerning candidate antigens for vaccine development, the use of transgenic mice for immune response research could help mapping responses to antigens and thus identifying more suitable vaccine candidates for humans. Transgenic mice expressing human HLA alleles have been used to study not only the binding of antigen epitopes but also T-cell responses [116]. HLA alleles influence disease outcomes and certain HLA types are associated with lower survival rates, indicating an impaired immune response [117]. Further, improvements in bioinformatics and in silico analysis tools should be exploited to facilitate the identification of previously overlooked antigen candidates or epitopes, which could be explored for vaccine design. Due to the high genetic diversity of *Burkholderia* isolates, establishing a pool of proteins shared by multiple strains (core proteome) could aid in broadening the pool of available antigens for cross-protection. As mentioned previously, experimental data from protection studies suggest that a multicomponent approach could improve the effectiveness of a subunit vaccine. DNA vaccine and viral vector vaccines are also promising strategies that merit further exploration since they have proven to be effective in eliciting protective immune response against diverse pathogens including bacteria, viruses, and parasites.

## Figures and Tables

**Table 1:** Inactivated whole-cell vaccines ^a^.

Species	Inactivation Method	Immunization Method	Challenge Strain	Challenge Method	Animal Model	Protection	Ref
*B. pseudomallei* K96243	Heat-killed	i.p. (10^8^ CFU)	*B. pseudomallei* K96243 *B. pseudomallei* 576 *B. pseudomallei* K96243 *B. mallei*	i.p. (3.5 × 10^5^ CFU) i.p. (2.2 × 10^4^ CFU) i.n. (92 CFU) i.n. (6.3 × 10^3^ CFU)	BALB/c mice	80% at day 21 100% at day 21 8 days (MTTD) 23.1 days (MTTD)	[26]
*B. pseudomallei* 576	Heat-killed	i.p. (10^8^ CFU	*B. pseudomallei* K96243 *B. pseudomallei* 576 *B. pseudomallei* K96243 *B. mallei*	i.p. (3.5 × 10^5^ CFU) i.p. (2.2 × 10^4^ CFU) i.n. (92 CFU) i.n. (6.3 × 10^3^ CFU)	BALB/c mice	100% at day 21 100% at day 21 9.75 days (MTTD) 13.78 days (MTTD)	[26]
*B. mallei*	Heat-killed	i.p. (10^8^ CFU)	*B. pseudomallei* K96243 *B. pseudomallei* K96243 *B. mallei*	i.p. (1.4 × 10^5^ CFU) i.n. (92 CFU) i.n. (6.3 × 10^3^ CFU)	BALB/c mice	70% at day 44 17.2 days (MTTD) 13.7 days (MTTD)	[26]
*B. thailandensis*	Heat-killed	i.p. (10^8^ CFU)	*B. pseudomallei* K96243 *B. pseudomallei* K96243 *B. mallei*	i.p. (1.4 × 10^5^ CFU) i.n. (92 CFU) i.n. (6.3 × 10^3^ CFU)	BALB/c mice	60% at day 44 7.4 days (MTTD) 6.3 days (MTTD)	[26]
*B. pseudomallei*	Heat-killed	i.n. (10^5^ CFU+ CLDC adjuvant)	*B. pseudomallei* 1026b	i.n. (7.5 × 10^3^ CFU)	BALB/c mice	55.5% at day 60	[31]
*B. pseudomallei* A2	Paraformaldehyde-killed Heat-killed	i.m. (10^8^ CFU)	*B. pseudomallei* A2	i.p. (100 CFU)	BALB/c mice	60% at day 30 All died within 6 days	[29]
*B. mallei*	Heat-killed, strain ATCC23344	s.c. (10^8^ CFU + Alhydrogel)	*B. mallei* ATCC23344	i.p. (2.3 × 10^8^ CFU)	BALB/c mice	All died within 21 days	[27]
*B. mallei*	Irradiation-inactivated, strain ATCC23344)	s.c. (10^8^ CFU + Alhydrogel)	*B. mallei* ATCC23344	i.p. (2.3 × 10^8^ CFU)	BALB/c mice	25% at day 2	[27]
*B. mallei*	Irradiation-inactivated, *B. mallei* capsule-negative mutant	s.c. (10^8^ CFU + Alhydrogel)	*B. mallei* ATCC23344	i.p. (2.8 × 10^8^ CFU)	BALB/c mice	All died within 21 days	[27]
*B. mallei*	Irradiation-inactivated, strain ATCC23344	s.c. (10^8^ CFU + Alhydrogel)	*B. mallei* ATCC23344 *B. mallei* (capsule-negative mutant) *B. mallei* (capsule-negative mutant)	i.p. (2.8 × 10^8^ CFU) i.p. (6.5 × 10^8^ CFU) i.p. (2.8 × 10^8^ CFU)	BALB/c mice	20% at day 21 100% at day 21 80% at day 21	[27]

^a^ Abbreviations: i.p., intraperitoneal; i.m., intramuscular; i.n., intranasal; i.v., intravenous; s.c., subcutaneous; CFU, colony forming units; MTTD, mean time to death; CLDC, cationic liposomes complexed with noncoding plasmid DNA.

**Table 2:** Live-attenuated vaccines ^a^.

Attenuated Strain	Immunization Method	Challenge Strain	Challenge Method	Animal Model	Protection	Ref
*B. pseudomallei* (Δ*ilvI*)	i.p. (10^6^ CFU)	*B. pseudomallei* 576 *B. pseudomallei* BRI	i.p. (10^6^ CFU)	BALB/c mice	80% at day 32 100% at day 32	[37]
*B. pseudomallei* (Δ*purN*)	i.n. (5 × 10^3^ CFU) i.n. (2 × 10^5^ CFU) i.p. (2 × 10^5^ CFU) i.n. (10^6^ CFU) i.p. (10^6^ CFU)	*B. pseudomallei* E8	i.p. (10^6^ CFU) i.n. (2 × 10^2^ CFU) i.v. (10^3^ CFU)	BALB/c mice	100% at day 9 37.5% at day 65 All died at day 65 All died at day 31 All died at day 27	[39]
*B. pseudomallei* (Δ*purM*)	i.n. (5 × 10^3^ CFU) i.n. (6 × 10^4^ CFU) i.n. (5 × 10^5^ CFU)	*B. pseudomallei* E8	i.p. (10^6^ CFU)	BALB/c mice	All died at day 7 All died at day 15 100% at day 17	[39]
*B. pseudomallei* (Δ*hisF*)	i.p. (7 × 10^3^ CFU)	*B. pseudomallei* E8	i.p. (10^5^ CFU)	BALB/c mice	50% at day 28	[39]
*B. pseudomallei* (Δ*pabB*)	i.p. (2 × 10^5^ CFU) i.p. (10^7^ CFU)	*B. pseudomallei* E8	i.p. (10^6^ CFU)	BALB/c mice	50% at day 65 75% at day 36	[39]
*B. pseudomallei* (Δ*aroB*)	i.n. (1 × 10^5^ CFU) i.n. (1 × 10^6^ CFU)	*B. pseudomallei* K96243	i.p. (5 × 10^4^ CFU) i.n. (1 × 10^3^ CFU)	BALB/c mice	All died at day 8 All died at day 8	[42]
*B. pseudomallei* (Δ*aroC*)	i.p. (3.5 × 10^7^ CFU) i.p. (5 × 10^8^ CFU)	*B. pseudomallei* A2 *B. pseudomallei* A2	i.p. (5 × 10^2^ CFU) i.p. (5 × 10^3^ CFU) i.p. (5 × 10^4^ CFU) i.p. (6 × 10^3^ CFU) i.p. (6 × 10^4^ CFU) i.p. (6 × 10^5^ CFU)	BALB/c mice C57BL/6 mice	All died All died All died 80% up to 5 months 60% up to 5 months 20% up to 5 months	[43]
*B. pseudomallei* (Δ*bipD*)	i.p.(10^4^ CFU)	*B. pseudomallei* 576	i.p. (10^4^ CFU)	BALB/c mice	60% at day 75	[46]
*B. pseudomallei* (Δ*asd*)	i.n. (1 × 10^7^ CFU)	*B. pseudomallei* 1026b	i.n. (4 × 10^3^ CFU)	BALB/c mice	All died at day 56	[44]
*B. pseudomallei* (Δ*relA* Δ*spoT*)	i.n. (1 × 10^5^ CFU)	*B. pseudomallei* 576	i.n. (1 × 10^3^ CFU)	C57BL/6 mice	60% at day 55	[45]
*B. mallei* (Δ*tonB*)	i.n. (1 × 10^2^ CFU) i.n. (1 × 10^3^ CFU) i.n. (1 × 10^4^ CFU)	*B. mallei* CSM001	i.n. (1.5 × 10^4^ CFU)	BALB/c mice	All died at day 15 62.5% at day 28 100% at day 28	[49]
*B. mallei* (Δ*tonB*Δ*hcp1*)	i.n. (1.5 × 10^5^ CFU)	*B. mallei* ATCC23344 *B. pseudomallei* K96243	i.n. (3.24 × 10^4^ CFU) aerosol (1.07–1.78 × 10^3^ CFU)	C57BL/6 mice	100% at day 21 87.5% at day 21	[51]
*B. mallei* (Δ*ilvI*)	Aerosol (7.3 × 10^4^ CFU)	*B. mallei* ATCC23344	i.n. (4.4 × 10^5^ CFU) i.n. (5 × 10^3^ CFU)	BALB/c mice	25% at day 30 50% at day 30	[47]
*B. mallei* (Δ*tssN*)	i.n. (prime, 1.3 × 10^5^ CFU); (boost, 2.3 × 10^4^ CFU)	*B. mallei* ATCC23344	i.n. (4.3 × 10^4^ CFU)	BALB/c mice	67% at day 30	[48]
*B. cenocepacia* (Δ*tonB*)	i.n. (5 × 10^7^ CFU)	*B. cenocepacia* K56-2 (Nx resistant mutant)	i.n. (5 × 10^7^ CFU)	BALB/c mice	87.5% at day 6	[21]
*B. mallei* (Δ*bat*)	i.t. (10^4^ CFU)	*B. mallei* ATCC23344 *B. pseudomallei* 1026b *B. pseudomallei* K96243	i.t. (8 × 10^3^ CFU) i.t. (2.5 × 10^4^ CFU) i.t. (2.5 × 10^4^ CFU)	BALB/c mice	56% at day 55 67% at day55 85% at day 55	[52]

^a^ Abbreviations: i.p., intraperitoneal; i.n., intranasal; i.v., intravenous; i.t., intratracheal; Nx, nalidixic acid; CFU, colony forming units.

**Table 3:** Subunit vaccines ^a^.

Species	Antigen	Adjuvant	Immunization Method	Challenge Strain	Challenge Method	Animal Model	Protection	Ref
*B. pseudomallei* K96243	LolC	MPL + TDM	i.p.	*B. pseudomallei* K96243	i.p. (4 × 10^4^ CFU)	BALB/c mice	83% at day 42	[60]
*B. pseudomallei* K96243	LolC	ISCOMS + CpG CpG MPL + TDM	s.c. s.c. s.c.	*B. pseudomallei* K96243	i.p. (7 × 10^4^ CFU)	BALB/c mice	33% at day 13 66% at day 13 50% at day 13	[60]
*B. pseudomallei* K96243	PotF	MPL + TDM	i.p.	*B. pseudomallei* K96243	(4 × 10^4^ CFU)	BALB/c mice	50% at day 42	[60]
*B. mallei* ATCC 23344	BopA BimA	ISCOM + CpG	i.n.	*B. mallei* ATCC 23344 (BmC) *B. pseudomallei* 1026b (BpC)	i.n. (10^3^ CFU)	BALB/c mice	100% at day 21 (BmC) 60% at day 50 (BpC) 100% at day 21 (BmC) 20% at day 50 (BpC)	[61]
*B. mallei* ATCC 23344	Hcp1	ISCOM + CpG	i.n.	*B. mallei* ATCC 23344 (BmC)	i.n. (10^3^ CFU)	BALB/c mice	78% at day 21 (BmC)	[61]
*B. pseudomallei* K96243	LolC	ISCOM + CpG	i.n.	*B. mallei* ATCC 23344 (BmC) *B. pseudomallei* 1026b (BpC)	i.n. (80 CFU)	BALB/c mice	82% at day 21 (BmC) 25% at day 50 (BpC)	[61]
*B. pseudomallei* D286	Omp85	Freund’s complete adjuvant/Freund’s incomplete adjuvant	i.p.	*B. pseudomallei* D286	i.p. (1 × 10^6^ CFU)	BALB/c mice	70% at day 15	[62]
*B. pseudomallei* K96243	Omp3 Omp7	Freund’s complete adjuvant/Freund’s incomplete adjuvant	i.p.	*B. pseudomallei* D286	i.p. (1 × 10^6^ CFU)	BALB/c mice	50% at day 21	[63]
*B. pseudomallei*	FliC	CpG	i.m.	*B. pseudomallei*	i.v. (1 × 10^5^ CFU)	BALB/c mice	93.3% at day 14	[64]
*B. pseudomallei*	OmpW	SAS	i.p.	*B. pseudomallei* 576	i.p. (4 × 10^6^ CFU) (6 × 10^5^ CFU)	C57BL mice BALB/c mice	75% at day 80 75% at day 21	[65]
*B. pseudomallei* 1026b	OMV		i.n. s.c.	*B. pseudomallei* 1026b	Aerosol (5.35 × 10^3^ ± 3.64 × 10^3^ CFU)	BALB/c mice	15% at day 14 60% at day 14	[66]
*B. pseudomallei* Bp82	OMV		s.c.	*B. mallei* China 7	Aerosol (1.246 × 10^3^ CFU) Aerosol (1.6 × 10^6^ CFU)	C57Bl/6 mice Rhesus macaques (*Macaca mulatta*)	80% at day 30 100% at day 21	[67]
*B. pseudomallei* K96243	Hcp 2 Hcp1 Hcp3 Hcp6 Hcp4	SAS	i.p.	*B. pseudomallei* K96243	i.p. (5 × 10^4^ CFU)	BALB/c mice	80% at day 42 50% at day 42 50% at day 42 50% at day 42 33% at day 42	[68]

^a^ Abbreviations: OMV, outer membrane vesicles; AuNP, gold nanoparticles; i.p., intraperitoneal; i.m., intramuscular; i.n., intranasal; s.c., subcutaneous; CFU, colony forming units. BmC B. mallei challenge; BpC B. pseudomallei challenge.

**Table 4:** Glycoconjugate vaccines ^a^.

	Conjugate	Adjuvant	Immunization Method	Challenge Strain	Challenge Method	Animal Model	Protection	Ref
*B. pseudomallei* K96243	CPS-Hcp1 CPS-CRM197 CPS-TssM	Alhydrogel + CpG	s.c.	*B. pseudomallei* K96243	Aerosol (≈4.65 × 10^7^ CFU/mL)	C57BL/6 mice	100% at day 35 67% 80%	[84]
*B. pseudomallei* K96243	CPS-LolC-BSA CPS-BSA	Alhydrogel + CpG	s.c.	*B. pseudomallei* K96243	i.p.	BALB/c mice	70% at day 35 50%	[85]
*B. pseudomallei*	CPS-TetHc	MPL/Sigma adjuvant system	i.p.	*B. pseudomallei* K96243	i.p. (1.02 × 10^5^ CFU)	BALB/c	67% at day 35	[86]
*B. mallei*	AuNP-FliC-LPS AuNP-Hcp1-LPS AuNP-TetHc-LPS	Alhydrogel	s.c.	*B. mallei* ATCC 23344	i.n. (2.27 × 10^5^ CFU) aerosol (5.0 × 10^9^ CFU)	BALB/c mice	60% at day 35 90% at day 35 70% at day 35	[89]
*B. mallei*	AuNP-FliC-LPS	Alhydrogel	s.c.	*B. mallei* ATCC 23344	aerosol (5.0 × 10^9^ CFU)	Rhesus macaques	50% at day 30	[87]
*B. pseudomallei*	TetHc-LPS		i.p.	*B. pseudomallei* K96243	i.p. (4.0 × 10^4^ CFU and 4.2 × 10^4^ CFU)	BALB/c mice	81% at day 29	[86]
*B. pseudomallei*	O-polysaccharide-AcrA	Imject Alum	i.p.	*B. pseudomallei* K96234	i.n. (2 × 10^3^ CFU)	BALB/c mice	40% at day 12	[93]

^a^ Abbreviations: OMV, outer membrane vesicles; AuNP, gold nanoparticles; i.p., intraperitoneal; i.m., intramuscular; i.n., intranasal; s.c., subcutaneous; CFU, colony forming units.

**Table 5:** DNA vaccines ^a^.

Antigen	Adjuvant	Immunization Method	Challenge Strain	Challenge Method	Animal Model	Protection	Ref
pcDNA-FliC	CpG ODN	i.m.	*B. pseudomallei*	i.v. (10^5^ CFU)	BALB/c mice	93.3% at day 12	[64]
pVAX-hTPA-FliC	Polyethylenimine	i.n.	*B. pseudomallei*	i.n. (500 CFU)	C57BL/6	53% at day 14	[103]

^a^ Abbreviations: i.m., intramuscular; i.n., intranasal; i.v., intravenous; CFU, colony forming units.

**Table 6:** Viral vector-based vaccines ^a^.

Antigen	Viral Vector	Immunization Method	Challenge Strain	Challenge Method	Animal Model	Protection	Ref
BatA	PIV5	i.n.	*B. mallei* ATCC 23344 *B. pseudomallei* K96243	Aerosol (8000 CFU) Aerosol (300 CFU)	BALB/c mice	74% at day 40 60% at day 35	[112]

^a^ Abbreviations: PIV5, Parainfluenza virus 5; i.n., intranasal; CFU, colony forming units.
